# Cocaine-Induced Isolated Bradycardic Atrial Flutter: A Rare Presentation in a Patient With a History of Acute Coronary Syndrome

**DOI:** 10.7759/cureus.43831

**Published:** 2023-08-20

**Authors:** Boran Mao, Abdul Jaraki

**Affiliations:** 1 Internal Medicine - Cardiology Rotation, St. George's University, Miami, USA; 2 Cardiology, Palmetto General Hospital, Miami, USA

**Keywords:** sotalol, bradycardia, african american male, acute chest syndrome (acs), cocaine use, atrial flutter

## Abstract

Six months ago, a middle-aged African American male visited the cardiology clinic for a follow-up on acute coronary syndrome along with atrial fibrillation. The patient was initially diagnosed with unstable angina with palpitation and underwent cardiac catheterization. During the visit, the patient complained of unspecific chest discomfort, palpitation, and reduced exercise tolerance after the use of cocaine for several months. ECG showed the absence of atrial fibrillation but instead showed atrial flutter with bradycardia. Cocaine-induced atrial flutter was suspected. The patient was educated about the imperative need to discontinue cocaine use immediately. Additionally, appropriate measures for rate control and anticoagulation were initiated.

## Introduction

Atrial flutter (AFL) is a type of supraventricular arrhythmia characterized by the presence of a re-entry mechanism that initiates tachycardia. For AFL to occur, patients must possess the following components: areas with fast and slow conduction velocities, different refractory periods, and a functional core where the circuit is present [1]. Risk factors include heart valve disorders, congenital heart diseases, coronary artery disease, high blood pressure, and overactive sympathetic activity. Patient symptoms can manifest in various ways but generally include palpitation, tachycardia, shortness of breath, dizziness, fatigue, syncope, and chest pain/discomfort.

Atrial fibrillation (AF) and AFL share similar risk factors and presentations, yet the occurrence of isolated AF or a combination of AF and AFL is significantly more prevalent than isolated AFL [2]. The treatment plan is the same for both conditions, aiming at controlling and reverting rate and rhythm to normal and preventing clot formation and embolization to prevent stroke-related disability.

There are two kinds of AFL, typical (80%) and atypical (20%). Typical AFL involves a single reentrant circuit with circus activation in the right atrium around the tricuspid valve annulus (most often in a counterclockwise direction), with an area of slow conduction located between the tricuspid valve annulus and the coronary sinus ostium (subeustachian isthmus). Typical counterclockwise AFL has caudocranial activation of the atrial septum (i.e., activation counterclockwise around the tricuspid valve annulus) [3].

Atypical AFLs may originate from the right atrium, as a result of surgical scars (i.e., incisional reentry), or from the left atrium, specifically the pulmonary veins (i.e., focal reentry) or mitral annulus. Left AFL is common and often problematic after left atrial linear ablation procedures (for AF). Thus, tricuspid isthmus dependency is not a prerequisite for atypical AFL [3].

In this study, we present a case of suspected cocaine-induced isolated AFL with typical major risk factors.

## Case presentation

A 57-year-old African American male, with a past medical history (PMH) of AF, essential hypertension (HTN), hypercholesterolemia, and hyperlipidemia (HLD), presented with intermittent palpitations, chest discomfort, and decreased exercise tolerance. He was visiting for a follow-up appointment related to a previous acute coronary syndrome (ACS) that had been managed with cardiac catheterization (Cardiac Cath) six months earlier. The patient initially reported experiencing gradual exertional retrosternal pressure, with chest pain radiating across the chest for five minutes. This discomfort was accompanied by feelings of dyspnea and palpitations. The symptoms subsided after five to seven minutes of rest. There was no associated trauma, fever, chills, nausea or vomiting, or diaphoresis. The exercise stress test with perfusion and echocardiogram was ordered, and a positive stress test was obtained. Unstable angina with AF was suspected, and Cardiac Cath was performed with no obvious obstruction of coronary vessels. The patient was controlled with medication and lifestyle modification. The patient reported recent and consistent use of cocaine over the past few months. The patient denied any other symptoms or complaints except those mentioned above.

Medication included rivaroxaban 20 mg oral tablet daily, simvastatin 10 mg oral tablet daily, and sotalol HCl 80 mg oral tablet BID (switched from the previous medication labetalol).

The vital signs indicated mild bradycardia at 56 beats per minute, and the patient was overweight, weighing 204 lbs. Physical examination showed diminished breath sound at bases with mild rhonchi, point of maximum impulse (PMI) in the fifth intercostal space 1 cm lateral to left midclavicular line, bradycardia, S1 greater than S2, A2 greater than P2, S4 present but no S3 and 2/6 systolic ejection murmur at the apex radiated to the axilla.

**Figure 1:**
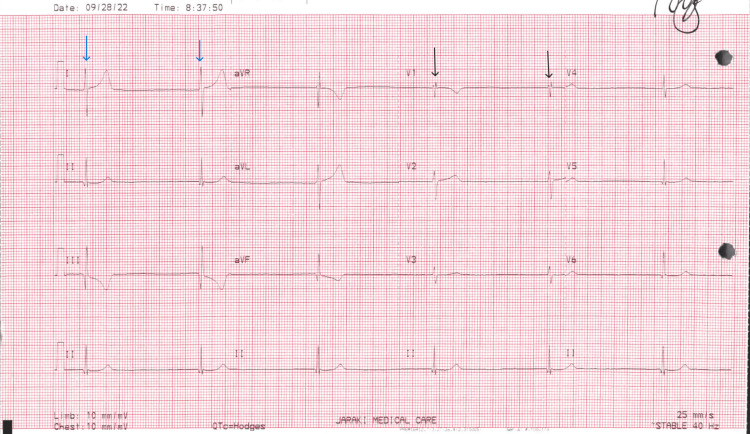
Post-Cardiac Cath four months ago with right axis deviation (blue arrows in Lead I showing negative QRS), right bundle branch block (black arrows in Lead V1 showing RSR’ pattern), old inferior infarct, and probable junctional rhythm with bradycardia of 34 beats per minute. Cardiac Cath, cardiac catheterization

A previous electrocardiogram (ECG) taken post-Cardiac Cath four months ago showed right axis deviation, right bundle branch block old inferior infarct, and probable junctional rhythm with bradycardia of 34 beats per minute (Figure 1).

**Figure 2:**
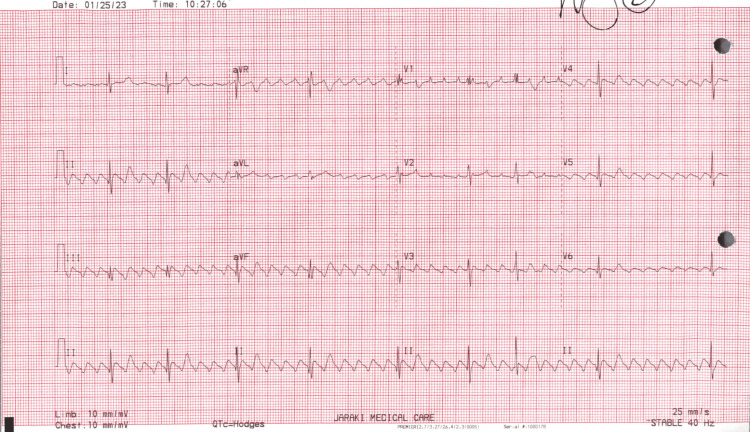
The latest ECG obtained during the office visit revealed multiple different ratios (3:1, 4:1, 5:1, and 6:1) indicative of atrial flutter with a heart rate of 56 beats per minute. ECG, electrocardiogram

A new ECG was performed that showed AFL with bradycardia of 56 beats per minute, variable atrial ventricular (AV) block, and possible old anterior infarct age undetermined, with the improvement of bradycardia but the presence of new AFL compared to post-Cardiac Cath ECG four months ago (Figure 2).

Treatment for AFL and bradycardia included continuing sotalol for stronger rate control, stopping any recreational drug use immediately to prevent worsening symptoms and future complications, and continuing the present anticoagulant regimen or adding a new anticoagulant to mitigate the risk of stroke or other embolic events. The patient would be advised to maintain a no-salt and low-fat diet, increase water intake, and incorporate moderate exercise as part of the HTN management plan. The patient would continue simvastatin along with diet and lifestyle modifications and rivaroxaban for six more months with regular outpatient follow-ups. If clinically appropriate, the patient may switch to aspirin in the future.

## Discussion

This report illustrated a rare development of isolated AFL triggered by cocaine usage. Cocaine is well known for its sympathomimetic effects of vasospasm and vasoconstriction, ischemic heart attack, and AF induction [4]. Our patient had HTN and ACS, which strongly increased the risk of developing both AFL and AF. For comparison with the current ECG, the ECG conducted four months ago after the Cardiac Cath procedure displayed right axis deviation, right bundle branch block, evidence of a previous inferior infarct, and a likely junctional rhythm accompanied by a bradycardic rate of 34 beats per minute. In response to this, the patient transitioned to taking sotalol twice daily and discontinued their initial use of labetalol.

It is relatively rare to see an isolated AFL induced by cocaine. The introduction of cocaine typically results in a higher likelihood of developing AF rather than AFL. Also, sotalol is a class III antiarrhythmic agent, which has several well-known side effects with prolonged use. Common side effects include QT prolongation, fatigue, bradycardia, chest discomfort, and abnormal ECG. Thus, it may be unclear whether the isolated AFL is caused by cocaine alone, sotalol alone, or both [5]. Comparing the previous ECG to the current ECG, sotalol provided a great improvement in heart rate from 34 to 56 beats per minute (compared to labetalol). However, the presence of AFL puts our patient at an increased risk of potential systemic embolization and stroke.

It is worth investigating the underlying cause of our patient's rare presentation of isolated AFL. While it is a rare presentation in this case, the patient had a couple of other risk factors that might contribute to isolated AFL development. In addition to cocaine, the patient is presently receiving treatment with sotalol, a recognized class III antiarrhythmic medication associated with potential side effects such as QT-interval prolongation and varying degrees of atrioventricular block. Sotalol may somewhat contribute to the development of AFL in the presence of cocaine. Furthermore, our patient previously had AF. With previous conduction abnormality, our patient is more likely to develop another conduction abnormality. Hence, further research and investigation will enable a better understanding of the process and can enhance patient management, education, and the selection of appropriate medications.

However, the effective treatment approach for AF/AFL remains consistent, encompassing both rate and rhythm control in addition to anticoagulation to mitigate the risk of potential stroke and associated disabilities [6]. Our patient was recovering well under sotalol; however, due to the presence of AFL, alternative medication options should be explored. Beta-blockers are generally a good option for rate control for AF/AFL. However, in our patient's case, due to a substantial bradycardic response to labetalol in the past, it might be necessary to explore alternative drug classes, such as non-dihydropyridine calcium channel blockers like verapamil and diltiazem. We may choose to continue the current medication treatment and conduct follow-up ECGs to determine whether sotalol plays a role in the development of AFL, or if it's solely due to cocaine. Additionally, we could consider a trial using different beta-blockers while closely monitoring the patient's response at frequent intervals [7]. Rivaroxaban should also be continued for embolization prophylaxis and management of previous ACS. Given our patient's HTN and hypercholesterolemia, it's essential to continue with diet and lifestyle modifications along with the ongoing use of statins for the long term. Furthermore, the consideration of catheter ablation is warranted, particularly due to its high efficacy and low complication rate in treating AFL. This option becomes even more relevant if our patient is refractory to medical treatment.

## Conclusions

Our patient was diagnosed with isolated, cocaine-induced AFL with a previous history of ACS and AF. In general, AF and AFL share several similar etiologies, risk factors, symptoms, and even complications. In this case, it may be worth investigating the underlying mechanism of isolated cocaine-induced AFL as it is a rare presentation affected by medication, social history, and PMH. Several risk factors could contribute to the development of isolated AF, and delving into the process and mechanisms through further investigation will enhance future management approaches for similar cases. However, the treatment plan is the same for both AF and AFL with anticoagulants and rate control agents. Patients with underlying heart disease should be educated on the importance of avoiding any recreational drug use, especially those with sympathomimetic properties. Regular follow-up and monitoring are essential to gauge improvements or deterioration in their condition, and any changes should be promptly addressed.
